# Programmable Droplet Microfluidics Based on Machine Learning and Acoustic Manipulation

**DOI:** 10.1021/acs.langmuir.2c01061

**Published:** 2022-09-13

**Authors:** Kyriacos Yiannacou, Vipul Sharma, Veikko Sariola

**Affiliations:** Faculty of Medicine and Health Technology, Tampere University, Korkeakoulunkatu 3, 33014 Tampere, Finland

## Abstract

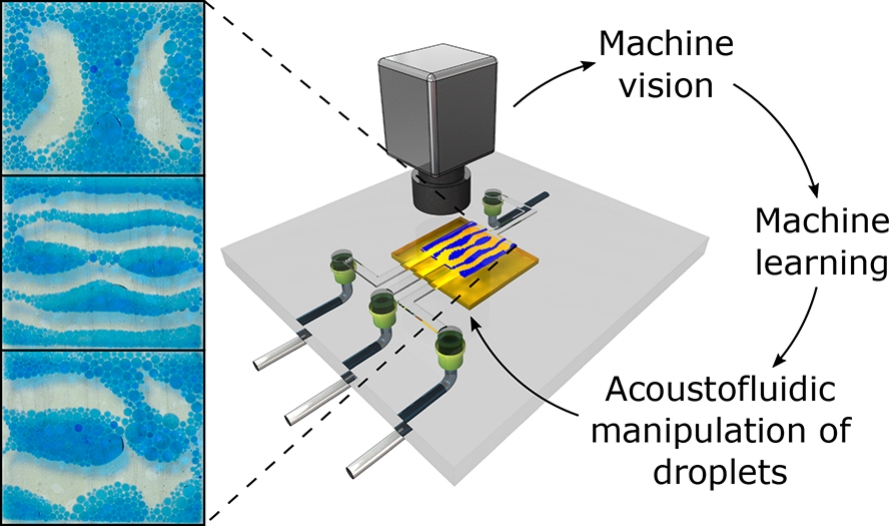

Typical microfluidic devices are application-specific and have to be carefully designed to implement the necessary functionalities for the targeted application. Programmable microfluidic chips try to overcome this by offering reconfigurable functionalities, allowing the same chip to be used in multiple different applications. In this work, we demonstrate a programmable microfluidic chip for the two-dimensional manipulation of droplets, based on ultrasonic bulk acoustic waves and a closed-loop machine-learning-based control algorithm. The algorithm has no prior knowledge of the acoustic fields but learns to control the droplets on the fly. The manipulation is based on switching the frequency of a single ultrasonic transducer. Using this method, we demonstrate 2D transportation and merging of water droplets in oil and oil droplets in water, and we performed the chemistry that underlies the basis of a colorimetric glucose assay. We show that we can manipulate drops with volumes ranging from ∼200 pL up to ∼30 nL with our setup. We also demonstrate that our method is robust, by changing the system parameters and showing that the machine learning algorithm can still complete the manipulation tasks. In short, our method uses ultrasonics to flexibly manipulate droplets, enabling programmable droplet microfluidic devices.

## Introduction

Designing a custom microfluidic chip for every application requires a considerable upfront investment, because each new chip design needs to be tested, iterated, and validated for the targeted application.^1,2^ Programmable microfluidic chips try to overcome this by offering reconfigurable functions, allowing the same chip to be used in multiple different applications.^2,3^ Currently, most programmable microfluidic devices are based on automating the operation of valves and pumps.^4−6^ However, tubes, valves, and pumps can quickly become the limiting factor in the miniaturization of microfluidic platforms,^7,8^ even if the chips themselves can be made very small.

As an alternative to mechanical pumps and valves, using acoustics has been proposed.^9^ In an acoustic microfluidic (*acoustofluidic*) chip, ultrasound transducers create acoustic waves, which can be used for transporting particles,^10−12^ cells,^13−15^ or droplets.^16−18^ More than just transporting, acoustics can also be used for sorting particles^10,19,20^ or droplets,^18^ merging droplets,^18,21,22^ and splitting droplets.^21−23^ Manipulating multiple solid objects by shaping the acoustic fields has been demonstrated, e.g., by using holographic acoustic traps^16,24,25^ or by using time-varying signals.^10,20,26^ Objects can be manipulated acoustically on the surface of the device or within a medium, using transverse waves,^21,26,27^ bulk acoustic waves,^10,18,28^ or surface acoustic waves.^7,11,22,29^

Droplets are a particularly attractive target for acoustofluidic manipulation. Using immiscible droplets in an inert medium as tiny carriers of samples and reagents is the goal of droplet microfluidics. The droplet volumes can be extremely small, ranging from a few nL to fL,^30^ saving chemicals,^30^ reducing reaction times,^30^ and allowing massively parallel operation.^31^ Typically, the droplets are water-based in an oil medium, and the droplets are produced with a flow-focusing device or a T-junction,^18,32^ where an immiscible continuous flow in a surrounding medium flow breaks into droplets. Droplets are a versatile medium for encapsulating and transporting cells, bacteria, or drugs, contributing to scaling down laboratory processes into miniaturized laboratories-on-chips, with studies ranging from genome sequencing,^33^ polymerase chain reactions,^34^ studying micro-organisms,^35^ high-throughput screening,^36,37^ and directed evolution.^38^ However, for a truly programmable device, it would be beneficial to be able to manipulate individual droplets: to transport them to any location inside the chip and to merge them at will.

Acoustics offers a natural path toward programmability: by changing the driving signals of the transducers (frequencies, amplitudes, phases, etc.), the shape of the acoustic field changes, and this changes how particles/droplets move inside the chip.^20,22^ Unfortunately, there is usually a complex relationship between the driving signal and the resulting acoustic field shape: the field shape is sensitive to small variations in chip dimensions and material properties.^39,40^ Using machine learning to predict the acoustic field shapes for a given driving signal has been proposed: machine learning algorithms can adapt to the nonidealities of an acoustic system.^10^

**Figure 1:**
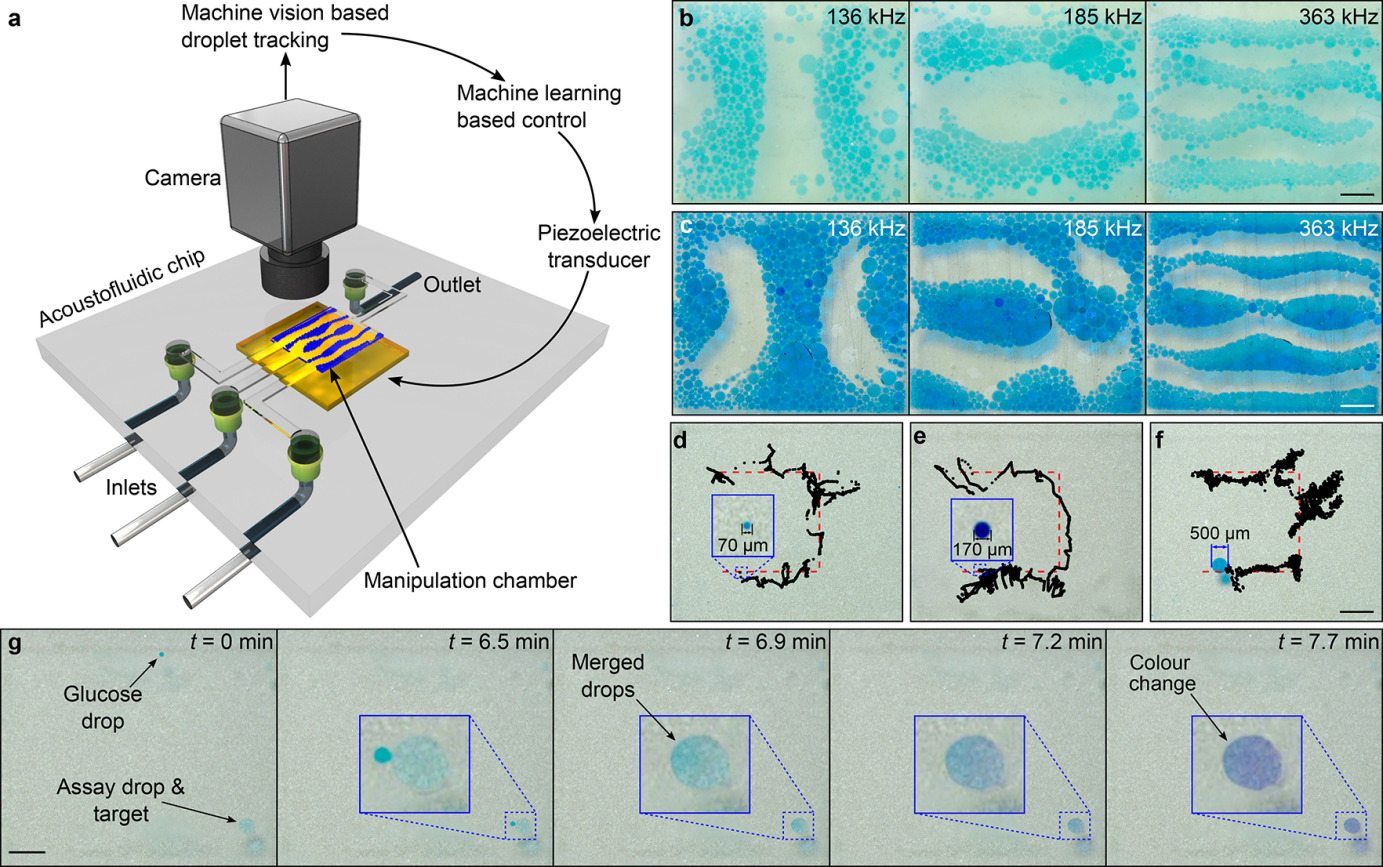
Acoustic transport and merging of droplets inside a microfluidic chip-based on machine learning. (a) Concept of the manipulation method. (b,c) Acoustic patterns of water droplets in oil (b), and oil droplets in water (c), for the frequencies 136 kHz, 185 kHz, and 363 kHz. The pattern formed by oil drops in water is the inverse of the pattern formed by water drops in oil, due to the opposite acoustic contrast factor. (d–f) Controlled manipulation of water droplets in oil, with varying droplet diameters: (d) 70 μm, (e) 170 μm, and (f) 500 μm. (g) Demonstration of a droplet-based glucose assay. A glucose droplet (dyed green) is guided toward a droplet containing the assay reagents (dyed light blue). The merging of the two droplets triggers a color change, turning the droplet purple. All scalebars are 1 mm.

In this paper, we report the programmable transport, merging, and mixing of multiple droplets inside acoustofluidic chips. Our approach is based on closed-loop machine vision tracking, a machine learning algorithm, and bulk acoustic waves, driven by a single piezoelectric transducer (Figure 1a). With our setup, we show that we can manipulate drops with volumes ranging from ∼200 pL up to ∼30 nL (Figure 1b–f). Unlike earlier work, we can freely position droplets in 2D and merge them at will. We demonstrate that a droplet containing reagents for a colorimetric glucose assay can be merged and mixed with a glucose-containing droplet, triggering a color change (Figure 1g). Finally, we show that the method is not limited to manipulating water droplets in oil (Figure 1b) but can also manipulate oil droplets in water (Figure 1c). In short, we propose using ultrasonics and machine learning for manipulating droplets, to enable programmable droplet microfluidic devices.

## Experimental Methods

The acoustofluidic manipulation takes place inside a microfluidic chip, fabricated by wet-etching fused silica glass. There is a rectangular manipulation chamber (width: 6 mm, length: 7 mm, height: 0.15 mm) in the chip. The chip has three inlets for pumping droplets containing different solutions and a single outlet leading to a collection reservoir. The inlets and the outlets have a width of 1.1 mm, and a depth of 0.15 mm. Fluidic connectors (Nanoports, IDEX Health & Science) were attached to the drilled inlets and outlet of the device. The microfluidic chip was fabricated by Klearia, France.

A piezoelectric transducer (NCE45, Noliac, Denmark) of dimensions 15 mm × 15 mm × 2 mm was glued by epoxy (Power Epoxy, Loctite) to the back of the chip. The transducer was driven with a signal from a computer-controlled arbitrary waveform generator (PCI-5412, National Instruments), amplified by a 400 W Class AB RF amplifier (1040L, Electronics & Innovation). The chip was imaged by a camera (acA2040–120uc, Basler, Germany) and illuminated by a 100 W LED panel. A personal computer was used to implement the closed-loop control. Three syringe pumps (Aladdin, World Precision Instruments) were used to pump the droplets in and out of the chip.

The acoustic manipulation of the droplets in our microfluidic chamber relies on the fact that the droplets have different acoustic properties compared to the medium. This difference is quantified by the acoustic contrast factor Φ,^41,42^ as given by
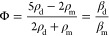
1where *ρ*_d,m_ are the densities of the droplet and the medium, and *β*_d,m_ are the compressibilities of the droplet and the medium. When the acoustic contrast factor is positive, the droplets move toward the pressure nodes (Figure 1b). When the contrast factor is negative, the droplets move toward pressure antinodes (Figure 1c). By using the material parameters from Table 1 and eq 1, we calculated the acoustic contrast factor for water droplets in oil (hexadecane) as Φ ≈ 0.766, implying that the water droplets will move toward pressure nodes. Similarly, we calculated the acoustic contrast factor for oil droplets in water as Φ ≈ −1.35, implying that the oil droplets will move toward the pressure antinodes.

**Table 1:** Properties of Fluids at 25 °C

Liquid	Density (kg m^–3^)	Speed of sound (m s^–1^)	Compressibility (GPa^–1^)
Hexadecane	770	1339^43^	0.864
Water	997	1496	0.415

The droplets were generated with an external flow-focusing device. The device channels have a height of ∼80 μm. The flow-focusing orifice has a width of 80 μm, and the two sheath flow inlets have a width of 150 μm. The schematic and the parameters of the flow-focusing device are summarized in Supplementary Figure S1a and Table S1. The flow focusing device was fabricated by using standard SU-8 photolithography (SU-8 2050, Microchem) to make a master mold and then by casting polydimethylsiloxane (Sylgard 184, 10:1 part A to B by weight, Dow Corning, USA) into the master mold.

For generating water droplets dispersed in the oil medium, we used ∼2.5 vol% sorbitan monooleate (Span 80, Sigma-Aldrich) in hexadecane oil (Fisher Chemicals) as the continuous phase and blue-dyed deionized water as the dispersed phase (droplets). Dyeing the droplets helped our machine vision algorithm to detect the droplets. Flow rates of *Q*_water_ = 0.5 μL min^–1^ and *Q*_oil_ = 50 μL min^–1^ generated water droplets with a diameter of approximately ∼70–80 μm (Figure 1d and Supplementary Figure S1b). Increasing the water flow rate while keeping the oil flow rate the same generated larger droplets: *Q*_water_ = 1 μL min^–1^ generated ∼100-μm-diameter droplets, and *Q*_water_ = 50 μL min^–1^ generated ∼200-μm-diameter droplets (Figure 1e). Water drops larger than 200 μm (Figure 1f) were produced by reducing sorbitan monooleate to 1.5 vol% and then by allowing smaller droplets to coalesce.

For generating hexadecane oil droplets dispersed in the water medium, we used 1 vol% of polyoxyethylene octyl phenyl ether (Triton X100, Sigma-Aldrich) and 15 g L^–1^ of sodium dodecyl sulfate (Sigma-Aldrich) in deionized water as the continuous phase and blue-dyed hexadecane oil (Fisher Chemicals) as the dispersed phase. Flow rates of *Q*_water_ = 50 μL min^–1^ and *Q*_oil_ = 5 μL min^–1^ generated oil droplets with a diameter of approximately ∼80–90 μm.

For the glucose assay (Figure 1g), we prepared a solution of 10 g L^–1^ of glucose in deionized water, and a solution containing the assay reagents: 1.8 g L^–1^ glucose oxidase, 1.6 g L^–1^ peroxidase, 0.6 g L^–1^ 4-amino antipyrine (4-AAP), and 1.36 g L^–1^*N*-ethyl-*N*-sulfopropyl-*m*-toluidine (TOPS) in phosphate-buffered saline (pH 7.4). All the chemicals were from Sigma-Aldrich. This chemistry was previously used as a basis of a colorimetric glucose assay in the work of Srinivasan et al.^44^ The glucose oxidase acts on the glucose to produce gluconic acid and hydrogen peroxide.^44^ The peroxidase acts on the hydrogen peroxide in the presence of 4-AAP and TOPS to produce a violet color quinonimine.^44^ The intensity of the violet color is directly related to the concentration of glucose: more glucose will lead to more hydrogen peroxide, and thus more quinonimine is produced. To generate the glucose and the reagent-containing droplets, we used the same flow rates as mentioned earlier for generating the water droplets. To make sure that the droplets containing the glucose and the assay reagents did not merge prematurely, they were pumped to the acoustofluidic chip through two different inlets.

The control algorithm used for the manipulation task is the ε-greedy algorithm from our previous work.^10^ Briefly, in each control cycle, machine-vision-detected positions of the droplets are provided to the control algorithm and its task is to choose which of the *N* discrete linearly spaced frequencies to apply next. Unless otherwise noted, *N* = 100, and the frequency range is from 65 kHz to 700 kHz. After the frequency is chosen, the piezoelectric transducer is excited at that frequency for half a second. The peak amplitude of excitation voltage was *U* = 17.6 V unless otherwise noted. After the excitation, the new positions of the droplets are detected, and the control algorithm assigns a reward for that frequency, the reward being simply how many micrometers the droplet moved toward the current target point. During the subsequent control cycles, the frequencies are chosen using the following heuristic: (1) with a probability 1 – ε, the algorithm chooses the frequency with highest average past reward; (2) otherwise (with a probability of ε), the frequency is chosen completely randomly. Throughout this paper, we used ε = 0.1. A simple explanation of the control algorithm is that it balances exploration and exploitation, exploiting most of the time frequencies that produced the best results in the past, but occasionally exploring new frequencies to see if they could produce even better results. The average rewards are weighted using exponentially decaying weights γ^*t*^, where γ = 0.9 is the weight factor and *t* is the number of control steps since that reward. Thus, recent observations carry a larger weight than past ones for the same frequency. More details on our control method can be found from our previous work^10^ and Supplemental Note 1.

## Results and Discussion

### Manipulation and Merging of Water Droplets in Oil

**Figure 2:**
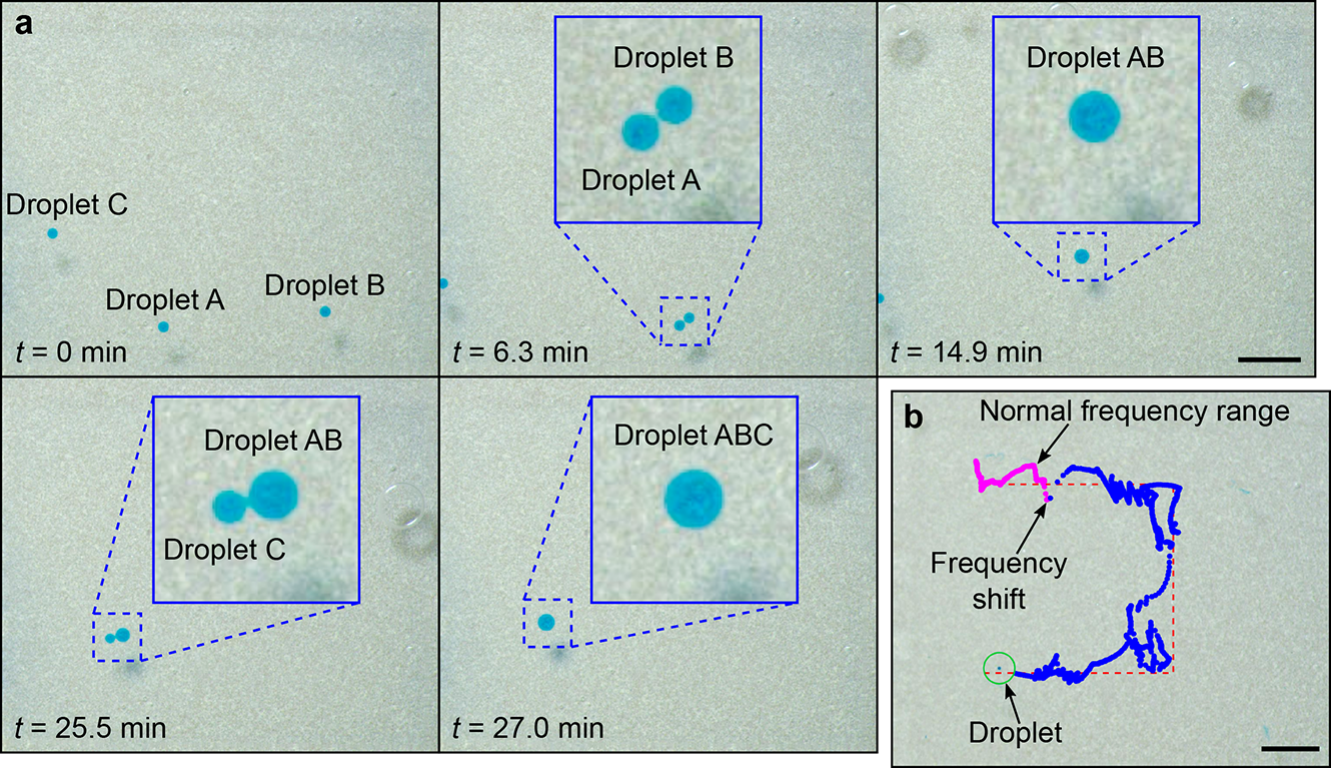
Transporting and merging water droplets in oil. (a) Two water droplets (droplet A and droplet B) were selected to merge at a specific target point, while droplet C position was uncontrolled. As soon as the two first droplets merged (droplet A and droplet B), the controller was switched to manipulating the remaining two droplets (droplet AB and droplet C) at a new target. (b) Effect of a frequency shift on the droplet manipulation. The controller guides a water droplet in oil through a planned path. Suddenly all the actuation frequencies are shifted by +10%, without hinting the controller about this change. All scale bars are 1 mm.

To show that we can actively manipulate and merge multiple water droplets in oil using our setup, we pumped three water droplets into the chamber and merged them. The merging was done by first actively controlling two of the drops (droplets A and B in Figure 2a) and guiding them close to each other. While the two droplets were being controlled, the third droplet (droplet C in Figure 2a) was allowed to passively transport in a semirandom manner. Next, ultrasonic pulses (2 s ON, 2 s OFF at 355 kHz, 112 V) were applied until the first two droplets merged (droplet AB in the Figure 2a). Finally, the remaining two droplets in the chamber were merged by using similar manipulation and pulsing steps. The whole experiment is shown in Figure 2a and Supplementary Movie S1. This experiment took a total time of 27 min.

When our controller brought two drops next to each other, the two droplets did not consistently merge, even if the droplets seemed to be in perfect contact, as viewed on the microscope image. We attribute this to the stabilizing effect of the surfactants on the interfaces of the droplets and to the low power of the ultrasound used during the manipulation. This was the reason that we used the high-power ultrasonic pulses to trigger the merging. Pulsing was used instead of continuous excitation to avoid possible heating of the chamber, although some heating of the medium was still observed (air bubbles appearing in the chamber). This highlights that the ultrasound can be used not only to transport the droplets, but also to disrupt the interfaces of droplets to merge droplets. In sum, the results in Figures 2a and Supplementary Movie S1 show that it is possible to transport and merge multiple droplets using our setup.

### Glucose Assay Chemistry in the Acoustofluidic Chip

To further show how this method could be adopted to a practical application, we performed the chemistry forming the basis of a colorimetric glucose assay in our acoustofluidic chip. In the experiment, two droplets—one with glucose as the analyte and another one with the assay reagents—are merged. The contents of the droplets interact and produce a color change, indicating the presence of glucose. The rate or the final degree of color change is related to the glucose content.^44^ The results are shown in Figure 1g and Supplementary Movie S2. As can be seen in the results, the controller successfully manipulated and merged the two drops within a period of 8 min. After merging, there is a significant color change of the mixed droplet to purple after ∼20 s, indicating the presence of glucose in the merged droplet. Overall, this experiment shows that we can use our setup to trigger specific reactions at will.

The previous experiment leaves open the question of whether unmerged droplets also would have changed their color if given enough time. To show that the color change occurs only when the two droplets merge, we performed an experiment where several glucose and reagent-containing droplets were pumped into the chamber and kept there without the presence of ultrasound. We recorded images of the drops for 24 h. Snapshots of this experiment are shown in Supplementary Figure S2. For the first 4.5 h of this experiment, we could not visually observe any significant color change in the droplets. After 24 h, we observed that some of the glucose droplets had started to change their color toward slightly darker hues. From the snapshots, we could not detect any reagent drops of appreciable size merging with the glucose droplets. This leaves two alternatives. Either there are small reagent droplets unobservable by our microscope that get transported and merged with the glucose drops, or some of the reagents from the reagent droplet slowly diffuse into the medium, eventually reaching the glucose droplets. Either way, we take this slow color change as evidence for the passive transfer of chemicals between droplets. However, whatever the reason for this passive transfer, this process is very slow: it takes significantly longer than our typical manipulation and merging experiments, which usually last tens of minutes to a maximum of an hour. We conclude that this passive transfer is unlikely to significantly affect the practical applications of our method.

### Manipulating Varying Sizes of Droplets

To test what different sizes of droplets can be manipulated with our setup, we pumped droplets of various diameters into the chip and tasked the controller to move the drops along a U-shape route. The diameters of the drops were ∼70 μm, ∼170 μm, and ∼500 μm, which correspond approximately to volumes of 200 pL, 3 nL, and 30 nL. For experiments with the smallest drops, the excitation voltage was increased to *U* = 20.6 V. The results are shown in Figure 1d–f and Supplementary Movie S3. All these different-sized water drops were successfully manipulated. The manipulation took 40, 66, and 60 min, respectively. These results offer further proof that our method is robust in manipulating different-sized droplets. Note that the depth of the manipulation chamber is only 150 μm, indicating that the two larger drops were already squeezed between the chamber floor and the ceiling. This did not cause the drops to stick to the glass, which we attribute to the stabilizing effect of the surfactants in the medium.

### Robustness of the Control Method to Changes in System Parameters

Acoustic fields are generally very sensitive to changes in the system: small cracks, delamination, heating, or bubbles could dramatically change how acoustic waves propagate in the system, potentially affecting the acoustic field shapes and modes of the system. The potential advantage of using machine learning algorithms that learn online is that they should be very robust to such changes: if a change occurs, the algorithm should be able to learn the new system parameters and still complete the manipulation. To demonstrate that our system has such robustness, we performed a transport experiment where the droplet was manipulated along a U-shaped path, but after 700 control steps, we suddenly shifted the actuation frequencies by +10%, without hinting the controller that this has happened. This frequency shift emulates a sudden change in system parameters—e.g., different fluids or a large bubble entering the chamber—which could change the resonances in the chamber. In these experiments, the excitation voltage was *U* = 42.2 V. The results are shown in Figure 2b and Supplementary Movie S4. As it can be seen from Figure 2b, the change in system parameters has little effect on our control method, and the control method quickly adapts to the new system parameters. We attribute this to the amnesiac nature of our control algorithm: thanks to the weight factor γ, the controller quickly forgets the past rewards and, by exploration, finds the new frequencies that move the droplet toward its target (Figure 2b). The experiment took 52 min, which is comparable to the time the experiment took without the frequency shift. In sum, the advantage of our amnesiac machine learning algorithm is that it is very robust to changes in system parameters.

### Robustness of the Controller to the Number of Manipulation Frequencies

The performance of the controller could be expected to be somewhat dependent on the number of discrete frequencies *N*. To test this, we performed the same manipulation experiment with a U-shaped path, but decreased *N*. We tested *N* = 30, *N* = 50, *N* = 75, and *N* = 100. The frequencies were reduced by dropping frequencies from the original set of *N* = 100 frequencies. Each experiment was repeated three times. The results are shown in Supplementary Figure S3. The controller completed the manipulation task, even when *N* = 30. There was no clear relationship between the number of frequencies to the time it takes to complete the manipulation. Overall, these results show that the method is not highly sensitive to the number of frequencies available to the controller.

### Controlled Manipulation and Merging Oil Droplets in Water

**Figure 3:**
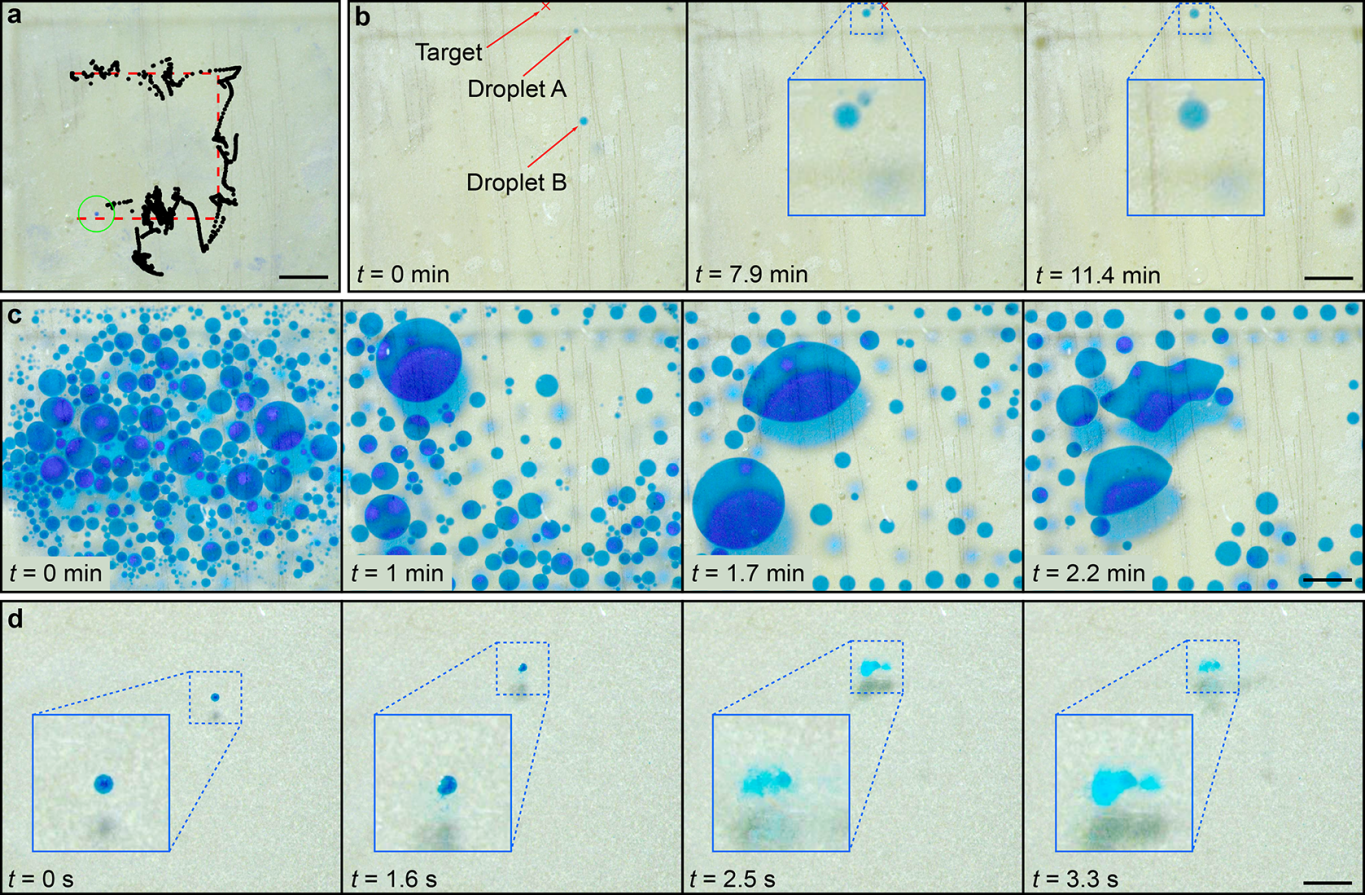
(a) Transport of hexadecane oil droplets in water medium. (b) Merging of two hexadecane droplets in water medium. (c) Many hexadecane droplets of various sizes in water medium. The droplets are coalescing in the presence of ultrasound, forming larger droplets. When the droplets become large enough relative to the wavelength, they start to deform in response to the acoustic field. (d) Ultrasonic disruption of a single water droplet in oil medium. A high-power ultrasound can disrupt the droplet interface, causing the droplet to split into multiple smaller droplets. All scale bars are 1 mm.

A more dramatic change in the manipulation is the reversal of the acoustic contrast factor (eq 1), which would result in the droplets transporting to antinodes instead of nodes. However, our control method makes no assumption of the droplets being transported either way, so it should be able to adjust even to such a dramatic change. To test this, we reversed the roles of oil and water in our system, by having hexadecane oil droplets dispersed in water medium. We first confirmed that the oil in water is transported to pressure antinodes, by comparing the Chladni pattern formed by both large numbers of water droplets in oil and large numbers of oil droplets in water. The results are shown in Figure 1b,c. The figures clearly show inverse Chladni patterns for the oil droplets in water, supporting the assumption that they get transported toward pressure antinodes. We then tasked the controller to manipulate an ∼80-μm-diameter oil droplet in water along the same U-shape path as before. The results are shown in Figure 3a and Supplementary Movie S5. In these experiments, the excitation voltage was *U* = 12.7 V. The manipulation was successful and took 38 min, which is again comparable to the experiments in manipulating water droplets in oil.

Next, we tested merging two oil droplets using our method. In these experiments, the excitation voltage was *U* = 14.1 V. The results are shown in Figure 3b and Supplementary Movie S6. To induce oil droplet merging, we had to increase the voltage: we applied 2-s-long pulses with an amplitude of 112 V at a frequency of 1.0087 MHz, followed by 2 s cool down. Pulsing was used instead of continuous excitation to avoid heating of the chamber, although some heating of the water medium was still observed. These higher voltages were sufficient to disrupt the interface and to merge the oil drops (Figure 3b).

To further study how the oil drops merge in the chamber, we pumped many different drops of various sizes to the chamber and applied the same 1.0087 MHz pulses as before, while observing the droplets coalesce. The results are shown in Figure 3c and Supplementary Movie S7. The results show that eventually drops start to coalesce, with most drops coalescing to one big drop. As a drop gets larger, it starts to significantly deform in the acoustic field, while smaller drops remain largely circular. We attribute this to two things: (a) the diameter of the droplet approaching the wavelength of the sound (1.3 mm in water and 1.2 mm in oil, at 1.0087 MHz, calculated using the values in Table 1) and (b) the Laplace pressure inside the droplet decreasing, as it is inversely proportional to the radius of the droplet.

In sum, the experiments with the oil droplets in water demonstrate that the control method can even adapt to the reversal of the acoustic contrast factor, showing that our method is not limited to only manipulating water droplets in oil.

### Ultrasonic Bursting of Droplets

If too high ultrasound power is applied, droplets can become unstable and burst. To demonstrate this, we pumped a single water droplet (radius: ∼100 μm) in oil medium, and scanned the frequencies in the usual frequency range 65 kHz to 700 kHz, but increased the voltage to 4.8 times as high, to 84 V. This means that the electric and acoustic powers are increased to ∼23 times as high. The results are shown in Figure 3d. At the frequency of 327 kHz, the droplet interface destabilized, causing the droplet to burst and split into multiple tiny drops. This phenomenon is related to the acoustic cavitation of gas bubbles in ultrasonic fields^45^ but with two immiscible fluids.^46^ This experiment highlights that there is a limit in the ultrasound power that can be applied during manipulation until the droplet interfaces are destabilized by the ultrasound.

## Conclusions

In this study, we propose that droplets inside a microfluidic device can be manipulated by using bulk acoustic waves from a single acoustic transducer, controlled by a machine learning algorithm. Both water droplets in oil and oil droplets in water could be manipulated and merged using the same device. Table 2 compares our results to some of the existing literature on manipulating immiscible droplets.

**Table 2:** Comparison of Our Results to the Reported Literature

Manipulation principle	Droplet and medium	Droplet diameter	Number of dimensions	Closed loop	ref
acousto-phoretic	oil in water, water in oil	70 μm–500 μm	2D	Yes	This work
	water in oil	2 mm	2D	No	(21)
	water in air	1 mm	2D	No	(47)
magneto-phoretic	ferrofluid in oil	170 μm–330 μm	2D	No	(48)
	ferrofluid in air	1.7 mm	2D	No	(49)
electro-phoretic	water in air	700 μm	2D	No	(50)
	double emulsions	1.6 mm	1D	No	(51)
dielectro-phoretic	water in oil	2 μm–30 μm	1D	No	(51)
	water in air	1 mm –3 mm	1D	No	(52)

Compared to the existing multidimensional acoustophoretic methods,^16,25^ the advantage of our method is that it is based on a single ultrasound transducer and not on multiple transducers or transducer arrays, simplifying the driving electronics, chip design, and fabrication. Another advantage of our method is that, due to its online machine learning nature, it can quickly adapt to changes in system parameters, as we have demonstrated. Further, we showed that with our method we can manipulate and merge droplets of various sizes, even drops greater than the depth of our chamber.

Compared to electrophoretic and magnetophoretic methods, our method has the advantage that it can produce complex field shapes even with a single transducer, whereas electrophoretic methods^50,53^ require careful placement of multiple electrodes, and magnetophoretic^54,55^ require close proximity to the coils for highly localized gradients. Furthermore, magnetophoretic methods require materials with specific magnetic properties, whereas our method relies on acoustic contrast and thus works with ordinary liquids, such as water and oil.

The downside of our method is that in its current incarnation, the manipulation times are relatively long: typically, a manipulation task can take from 10 min to an hour. As a future work, the controller should be improved and sped up. A straightforward approach would be to shorten the actuation time per cycle from the half a second which it is currently. The image acquisition and the machine-vision-based particle tracking are the bottlenecks. However, high-speed closed-loop acoustic manipulation of particles on a Chladni plate has been previously demonstrated,^26^ so we do not think the particle tracking speed is a fundamental limitation of our method. Another way to possibly improve our method is to increase the actuation voltages, as the droplets should move faster in high power acoustic fields. However, we already showed that the voltage cannot be increased too much, as high voltages caused the droplet to burst.

In future work, one potential way to dramatically increase the throughput of our method is by massively parallelizing the manipulation: using cameras with bigger sensors to track thousands of droplets, GPU accelerated computing for detecting the droplets, and multiple transducers or transducer arrays to shape the field for manipulating multiple particles at the same time. In principle, the machine learning algorithms can be relatively easily adjusted to control multiple transducers instead of just one, without significantly changing the fundamental working principle of the algorithm.

In addition to throughput, the precision of our manipulation method should also be improved: the results in Figure 1d–f show that there was considerable amount of randomness in the paths taken by the droplets. This is mainly due to the ε-greedy control algorithm: the algorithm only learns which frequencies are good locally, depending on the current position of the droplet. As soon as the droplet moves sufficiently that the local field shapes have changed, the algorithm forgets how the droplet moved in its previous position and learns how the droplet moves in its new position. If the droplet returns to a position where it has been before, the controller has forgotten everything about the original position and has to relearn everything again. This is a very robust controller, as it cares little for changes in the physical system, but the downside of the ε-greedy controller is the poor precision, as it needs to “re-explore” the frequencies at each position. A better controller would remember how the droplet moved at each position, and when the droplet returns to a position where it has been earlier, the controller could already use its model to choose which frequencies are likely to be good. However, the controller should not became overconfident in its model, as the acoustic parameters of the chamber could suddenly change (e.g., a bubble inside the chamber). Future work should explore what type of models are the best specifically for this type of acoustic manipulation, and what is the best balance between model confidence, complexity, and adaptivity.

One of the disadvantages that arose only when manipulating oil droplets in water and not when manipulating water droplets in oil was that in the former case, the oil droplets get transported toward the antinodes which exist always near the chamber walls. Thus, there is a risk that the oil droplets get trapped to the walls. This could be tackled by making sure all the manipulation target points are sufficiently far away from the chamber walls, or by using syringe pumps to induce a fluid flow to drag the droplets away from the walls.

To conclude, our acoustophoretic manipulation of droplets offers a natural path toward reprogrammable microfluidic platforms, as the sequence of transporting and merging fluids can be controlled by reprogramming the control task. We expect this to prove particularly useful for applications where a large number of different chemical reactions are needed in small volumes, e.g., lab-on-a-chip-based drug discovery or bioassay test panels.

## Supporting Information Available

CAD designs and images of the flow focusing device. Images and histograms of color changing glucose droplet. Scatter graph with manipulation times and Images of droplet manipulations, for the experiments with altered number of frequencies. Table containing the designing parameters of the flow focusing device. ε-greedy algorithm formulations. Full descriptions of the supplementary movies. (PDF)
Supplementary Movie S1: A movie showing merging of three water droplets in oil (MP4)
Supplementary Movie S2: A movie showing merging of a colorimetric glucose assay drop and a glucose droplet, resulting in color change (MP4)
Supplementary Movie S3: A movie showing manipulation of different sized water droplets (MP4)
Supplementary Movie S4: A movie showing water droplet manipulation with a sudden frequency shift (MP4)
Supplementary Movie S5: A movie showing oil droplet manipulation (MP4)
Supplementary Movie S6: A movie showing manipulation and merging of oil droplets (MP4)
Supplementary Movie S7: A movie showing coalescing of oil droplets (MP4)
