# Supplementary material for: Programmable Droplet Microfluidics Based on Machine Learning and Acoustic Manipulation
Source: Langmuir. 2022 Sep 13;38(38):11557–64. doi: 10.1021/acs.langmuir.2c01061 (PMC9520974; doi:10.1021/acs.langmuir.2c01061)
Supplement: Supplementary file 1 — la2c01061_si_001.pdf [file la2c01061_si_001.pdf]

Programmable Droplet Microfluidics Based on Machine Learning and Acoustic Manipulation

*Kyriacos Yiannacou, Vipul Sharma and Veikko Sariola**

Faculty of Medicine and Health Technology, Tampere University, Korkeakoulunkatu 3, P.O. Box 692, 33014 Tampere University, Finland

* veikko.sariola@tuni.fi

Supporting Information

Supplementary Figures

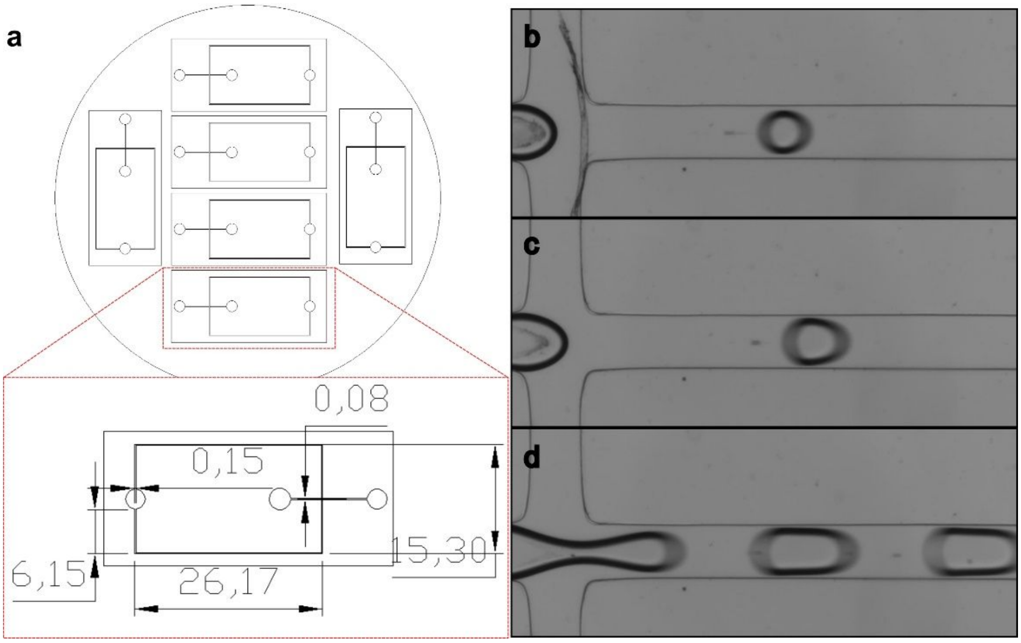

Figure S1. a) Schematic diagram of the flow focusing device used for generating water and oil droplets. The designing parameters of the flow focusing device were calculated based on the parameters found in the work of Lashkaripour et al.[1] These details are summarized in the Table S1. **b-d)** Microscope images of the flow focusing device when operated at different flowrates to generate different sized droplets.

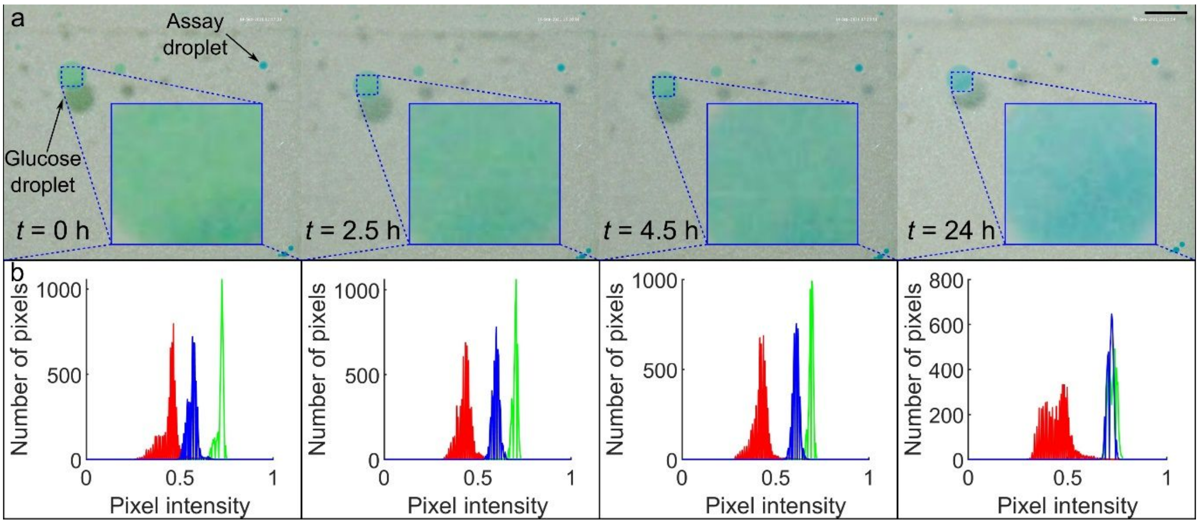

Figure S2. **a)** Snapshots of glucose and enzyme containing droplets in the acoustofluidic chamber without the presence of ultrasonic excitation. **b)** All histograms are calculated based on the area of the large droplet shown in the inset for all timeframes. Scalebar is 1 mm.

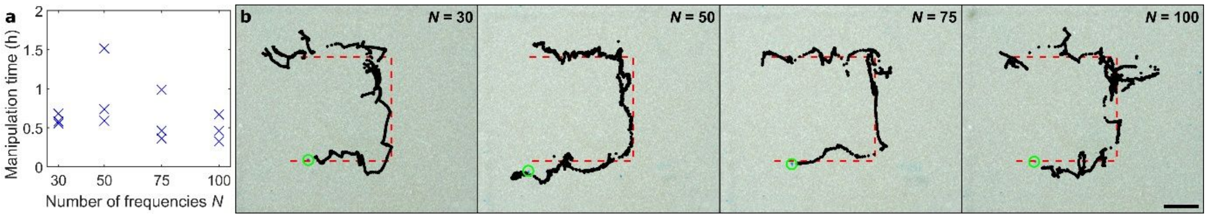

Figure S3. **a)** Manipulation time for water droplets in hexadecane for different number of frequencies *N*. The experiment for each *N* was repeated three times. **b)** The manipulation paths for one of the experiments in a) for each *N*. Scalebar: 1 mm.

Supplementary Table

Table S1. Flow-focusing geometry parameters, following the notation of Lashkaripour et al.[1]

Name	Symbol	Value
Orifice Width (μm)	Or.	80
Aspect Ratio	A.R.	1
Expansion Ratio	Λ	2
Normalized Water Inlet	Wc	2
Normalized Oil Inlet	Wd	2
Normalized Orifice Length	$Or._L$	1

Supplementary Note

Pseudocode for the multiarmed bandit control algorithm

In the following, N is the number of discrete frequencies used for the manipulation. In our experiments, the hyper parameters below were chosen as $N = 100$, $\epsilon = 0.1$, and $\gamma = 0.9$ unless otherwise noted. These hyper parameters were chosen based on initial exploration as they resulted in successful manipulation. The algorithms are given here in recursive form. The algorithm is same as one of the algorithms we used in our previous work.[2]

The ε-greedy control algorithm with decaying memory

Parameters: $\varepsilon \in (0,1]$ and $\gamma \in (0,1]$

Initialization: $w_i(0) = \mu_i(0) = 0$ for $i = 1 \ldots N$

For each control step $t = 1 \ldots$

If any of the frequencies has not been chosen (i.e., $w_i(t-1) \ = \ 0$)

$$\pi(t) = i$$

Else with the probability ε:

$$\pi(t) = \text{ randomly from the range } 1 \ldots N$$

Else:

$$\pi(t) = \arg\max_{\mathrm{i}} \mu_i(t-1)$$

Set $d_k(t)$ as the distance of droplet k from its target point (detected using machine vision)

Apply frequency number $\pi(t)$

Set $\tilde{d}_k(t)$ as the distance of droplet k from its target point (detected using machine vision)

Calculate reward: $r(t) = \sum_k \tilde{d}_k(t) - d_k(t)$

Decay weights: $w_i(t) = \begin{cases} \gamma w_i(t-1) + 1 & \text{if } i = \pi(t) \\ \gamma w_i(t-1) & \text{otherwise} \end{cases}$

Update mean: $\mu_{\pi(t)}(t) = \mu_{\pi(t)}(t-1) + (r(t) - \mu_{\pi(t)}(t-1))/w_{\pi(t)}(t)$

Supplementary Movies

Supplementary Movie S1. Merging of multiple water droplets in the acoustofluidic chamber. Three water droplets are merged sequentially in the acoustofluidic chamber. Firstly, the controller guides the water droplets A and B at the selected target point, while the third droplets move in a random fashion. Then, high energy ultrasound pulses are applied to trigger the merging of the drops. Next, the droplet AB is merged with droplet C using similar control and pulsing steps.

Supplementary Movie S2. Colorimetric glucose assay. The movie shows an experiment, where two droplets—one with glucose and another one with the assay reagents—are merged, and their content interacts, producing a color change indicating the presence of glucose.

Supplementary Movie S3. Manipulation of droplets of different sizes in the acoustofluidic chamber. The controller is tasked to transport droplets of sizes 70 μm, 170 μm and 500 μm through a predefined route forming a U-shaped path.

Supplementary Movie S4. Manipulation of water droplet through a U-shaped path when after 700 control steps the frequency range was shifted by +10%.

Supplementary Movie S5. Transportation of a Hexadecane oil droplet through a U-shaped path.

Supplementary Movie S6. Oil droplet merging in water. Two hexadecane droplets are transported at a specific location and merged. After manipulation, high energy ultrasound pulses are applied to trigger the merging of the drops.

Supplementary Movie S7. Coalescence of oil droplets in water. Smaller droplets of hexadecane oil coalesce in the presence of ultrasound, forming larger droplets.

Reference.

(1) Lashkaripour, A.; Rodriguez, C.; Ortiz, L.; Densmore, D. Performance Tuning of Microfluidic Flow-Focusing Droplet Generators. *Lab Chip* **2019**, *19* (6), 1041–1053. https://doi.org/10.1039/C8LC01253A.

(2) Yiannacou, K.; Sariola, V. Controlled Manipulation and Active Sorting of Particles Inside Microfluidic Chips Using Bulk Acoustic Waves and Machine Learning. *Langmuir* **2021**, *37* (14), 4192–4199. https://doi.org/10.1021/acs.langmuir.1c00063.
